# Women’s Perception and Attitude Towards Using Antidepressants During Pregnancy: A cross sectional study

**DOI:** 10.1192/j.eurpsy.2022.2203

**Published:** 2022-09-01

**Authors:** S. Alshahrani

**Affiliations:** King Abdullah bin Abdulaziz University Hospital, Princess Nourah bint Abdulrahman University, Department Of Neurosciences, Riyadh, Saudi Arabia

**Keywords:** Depression, antidepressant, Pregnancy, Antidepressants

## Abstract

**Introduction:**

Depression during pregnancy leads to deterioration of the mothers’ and the fetus’ health.

**Objectives:**

To explore women’s perception and attitude towards using antidepressants during pregnancy and identify the factors that influence decision making regarding antidepressants use.

**Methods:**

A cross-sectional survey of 991 subjects using convenient sampling. All study subjects (PNU affiliates; staff and students) were invited to fill out an electronic questionnaire, KAAUH staff and PNU female associates who were less than 18 years old were excluded. Answers were reported using 5- point Likert scale. The responses were summed up to give a total score for each respondent. The cutoff point is 75%. Respondents who scored above or equal 75% of the total score was considered as positive perception or favorable attitude.

**Results:**

The majority of women had negative perception and favorable attitude towards using antidepressants during pregnancy reaching 64%. While, women with positive perception and favorable attitude represented about 20% of the study subjects. The main factors influencing decision making were, education specialty (health, none-health) and subject history of diagnosis with any psychological disorder. Social stigma, religious believes and fear of addiction were reported by surveyors to be the reason influencing their perception and attitude about antidepressants use (P value <0.005).

**Conclusions:**

This study reveals that although Saudi women reflect a negative perception towards using antidepressants during pregnancy, yet they have a favorable attitude once depression during pregnancy becomes an issue.

**Disclosure:**

No significant relationships.

